# Mycobacterium tuberculosis Complicating an Elective Right Total Knee Arthroplasty

**DOI:** 10.7759/cureus.101774

**Published:** 2026-01-18

**Authors:** Edward Eusanio, Brian Shaw, Mario Madruga, Melinda Madden, Steve Carlan

**Affiliations:** 1 Internal Medicine, Orlando Regional Medical Center, Orlando, USA; 2 Infectious Diseases, Orlando Regional Medical Center, Orlando, USA; 3 Academic Affairs and Research, Orlando Regional Medical Center, Orlando, USA

**Keywords:** infected knee arthroplasty, multidrug-resistant (mdr) tb, mycobacterium tuberculosis complex, osteoarthritis, prosthatic joint infection

## Abstract

*Mycobacterium tuberculosis* primarily causes pulmonary disease but can involve extrapulmonary sites, including bones and joints. Tuberculosis (TB) prosthetic joint infections (TB-PJIs) are rare, often diagnosed late due to low suspicion, indolent progression, and frequent culture negativity.

A 73-year-old man from Guyana underwent elective right total knee arthroplasty for osteoarthritis. Postoperatively, he developed a prosthetic joint infection without an identifiable pathogen by routine cultures. Advanced testing detected *Mycobacterium tuberculosis* complex via next-generation 16S rRNA gene sequencing. The patient initially improved with RIPE (rifampin, isoniazid, pyrazinamide, and ethambutol) therapy but subsequently developed disseminated disease, raising concern for suspected drug-resistant TB. His hospital course was further complicated by bladder rupture and multiorgan failure, leading to death.

TB-PJIs may present without pulmonary symptoms or prior TB history and should be suspected in culture-negative joint infections, particularly when conventional therapy fails. Treatment may be further complicated by suspected multidrug resistance, as illustrated in this case.

## Introduction

Tuberculosis (TB) remains a leading cause of global morbidity and mortality, with an estimated 10.8 million cases in 2023, approximately 3.2% of which occurred in the Americas [1]. While TB most commonly affects the lungs, extrapulmonary manifestations occur in about 20% of cases [2], with musculoskeletal involvement accounting for 1-3% [3]. Vertebral TB (Pott’s disease) is the most common form, followed by arthritis, usually involving weight-bearing joints [4]. Prosthetic joint infections (PJIs) caused by* Mycobacterium tuberculosis* are exceedingly rare, comprising only 0.2% of all culture-positive PJIs [5,6]. Diagnosis is often delayed due to nonspecific symptoms, cultural limitations, and co-infections with more common organisms [7]. This case highlights the challenges of diagnosing and managing a TB-PJI, especially in a patient without a known TB history.

## Case presentation

A 73-year-old male with a medical history including hypertension, gout, type 2 diabetes mellitus, myelodysplastic syndrome, asthma, and osteoarthritis presented to the hospital for a right total knee arthroplasty for severe pain and disability caused by osteoarthritis. He was originally from Guyana and denied any history of TB infection.

**Figure 1:**
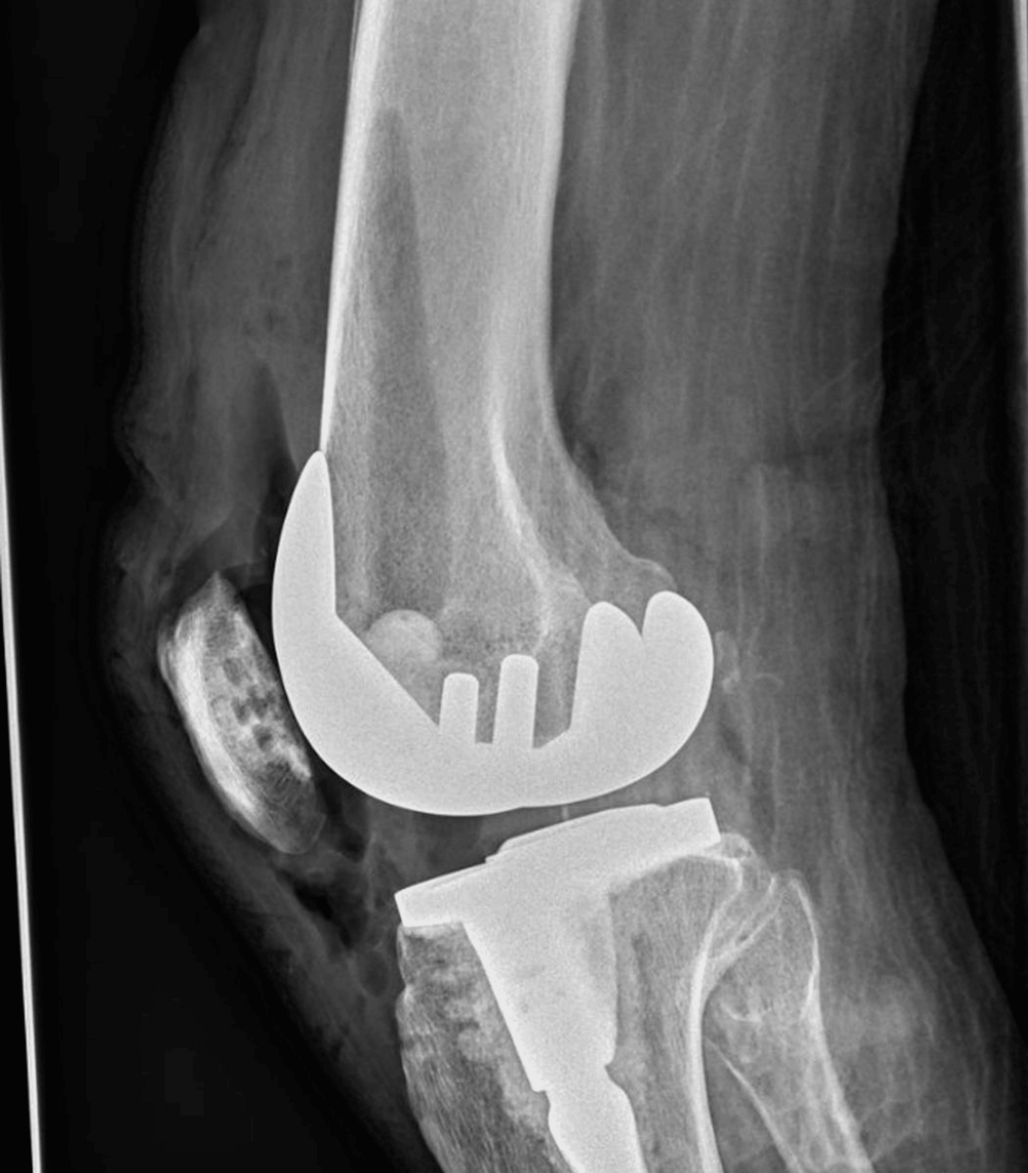
X-ray of the right knee after initial right total knee arthroplasty showing expected postsurgical changes.

The patient tested negative for HIV (human immunodeficiency virus) and was not on steroids. The surgery was completed without intraoperative complications. Early in the postoperative period, he developed worsening leukocytosis and fevers; a knee X-ray was performed, which was negative (Figure 1).

**Figure 2:**
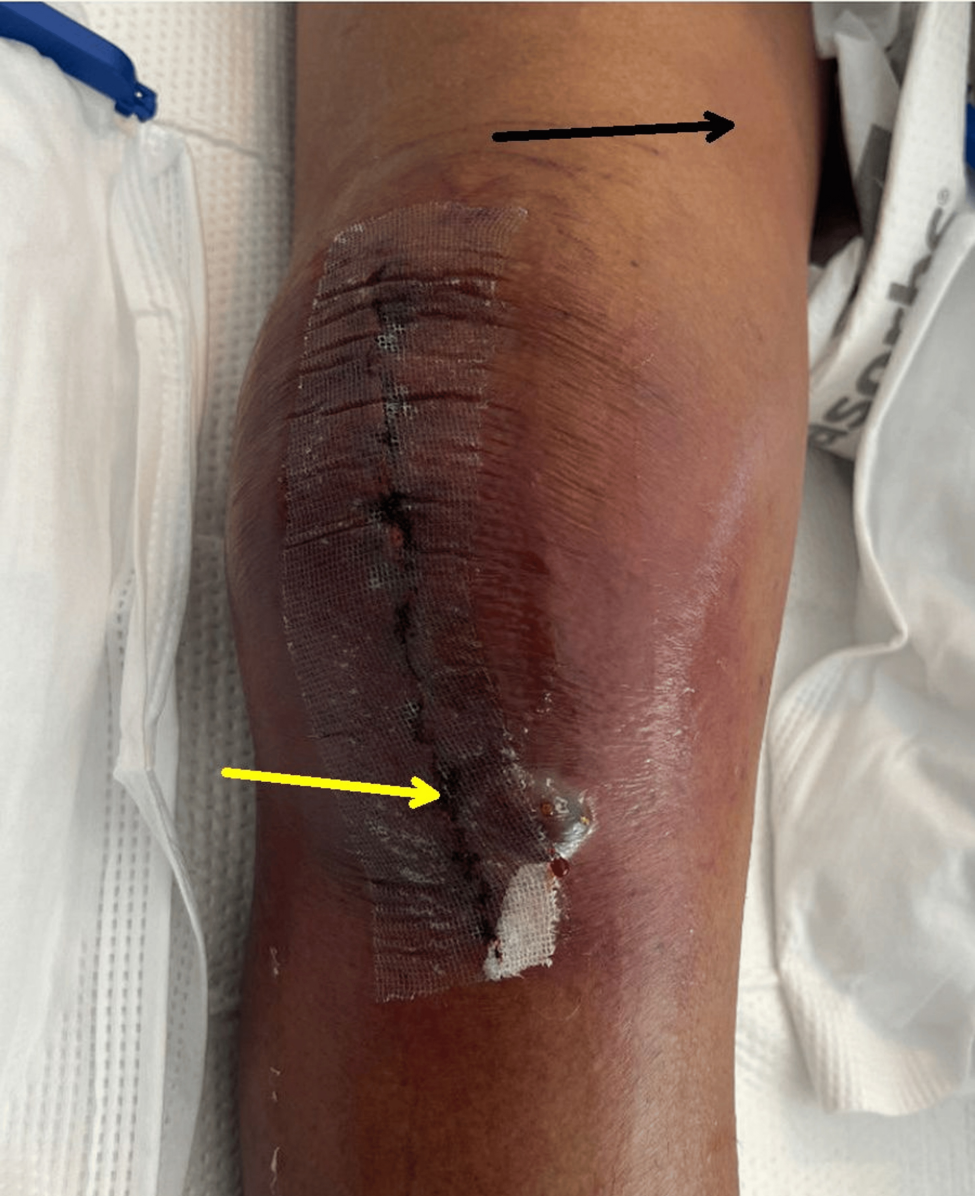
Right knee on postoperative day 5 with dusky erythema with bulla (yellow arrow) adjacent to the incision and erythematous streaking/lymphangitis up the thigh (black arrow).

He was started on broad-spectrum, empiric antibiotics including daptomycin, clindamycin, and cefepime. On postoperative day (POD) 5, the right knee appeared dusky with erythema, a bulla near the incision, and erythematous streaking/lymphangitis up the thigh (Figure 2).

**Figure 3:**
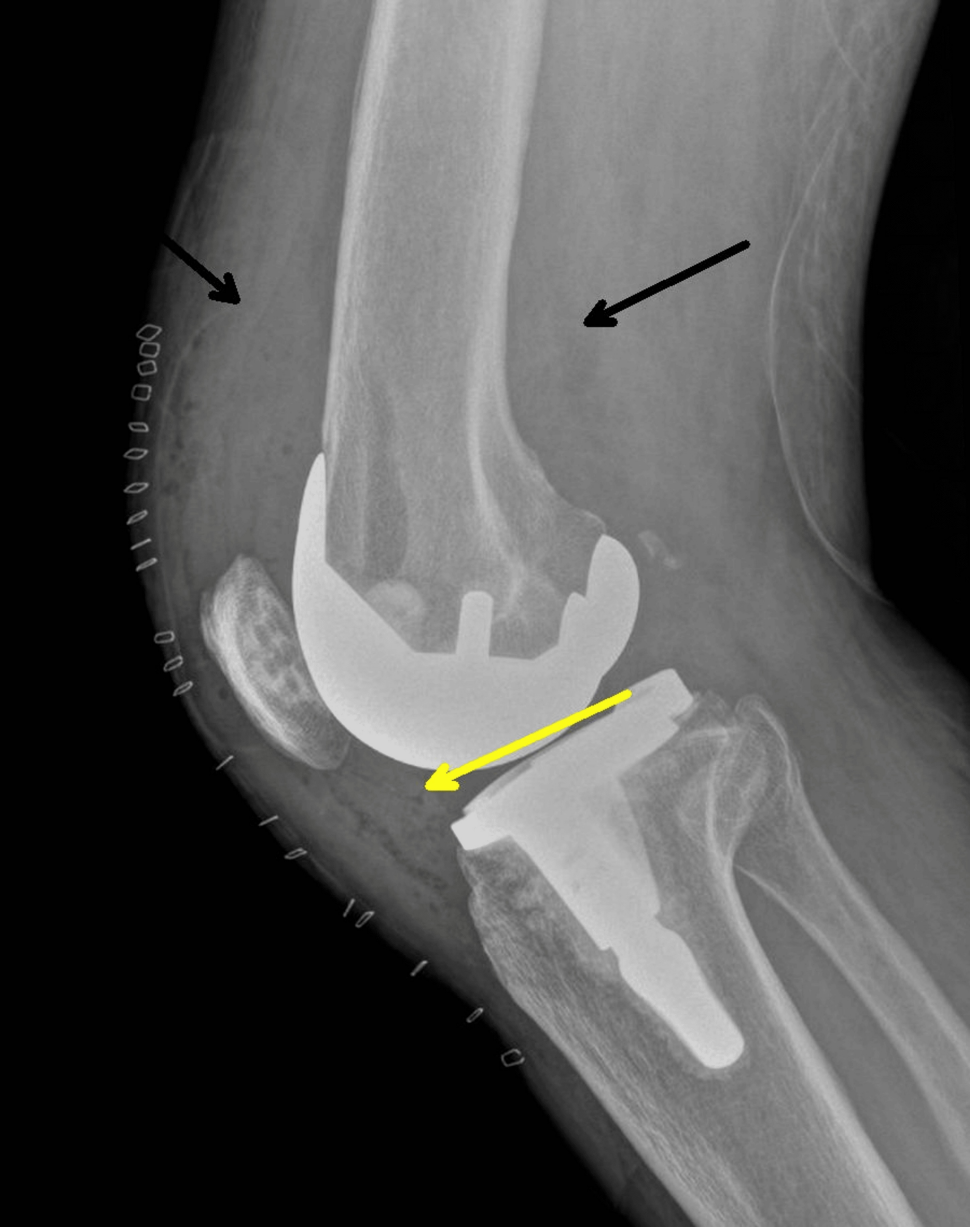
Right knee X-ray six days after debridement and polyethylene exchange. It shows an intact prosthesis along with soft tissue swelling (black arrows) and a moderate-sized joint effusion (yellow arrow).

A joint aspiration demonstrated 25 mL of bloody aspirate with 2,455,000 red blood cells/uL (reference range: <2,000/mm^3^ (0 to 2000/mm^3^), 75,5500 white blood cells/uL (reference range: <200 cells/cubic millimeter (mm^3^) (0 to 200/mm^3^), and 98% neutrophils (reference range: <25%) but was otherwise inconclusive. The infectious workup, including blood and synovial fluid cultures, was negative. On POD 6, the patient underwent irrigation and debridement of the right knee arthroplasty with polyethylene spacer exchange, and a right knee X-ray showed a moderate-sized joint effusion (Figure 3). 

Cultures taken during the procedure did not grow any organisms, so next-generation sequencing was pursued due to persistently negative synovial and operative cultures in the setting of a suspected PJI, in accordance with institutional infectious disease practice. Next-generation 16S rRNA gene sequencing from tissue was positive for *Mycobacterium tuberculosis* complex several days later. The patient was started on RIPE (rifampin, isoniazid, pyrazinamide, and ethambutol) therapy and showed clinical improvement. Tests including Quantiferon Gold, acid-fast bacilli sputum cultures, and Karius testing were all negative during hospitalization. The patient also underwent bronchoscopy with bronchoalveolar lavage, which also yielded negative results for acid-fast bacilli (AFB) and cultures. After a 30-day hospital stay, he was discharged to a skilled nursing facility on RIPE therapy, with follow-up at the Department of Health and infectious disease clinic, where he initially improved over several weeks.

**Figure 4:**
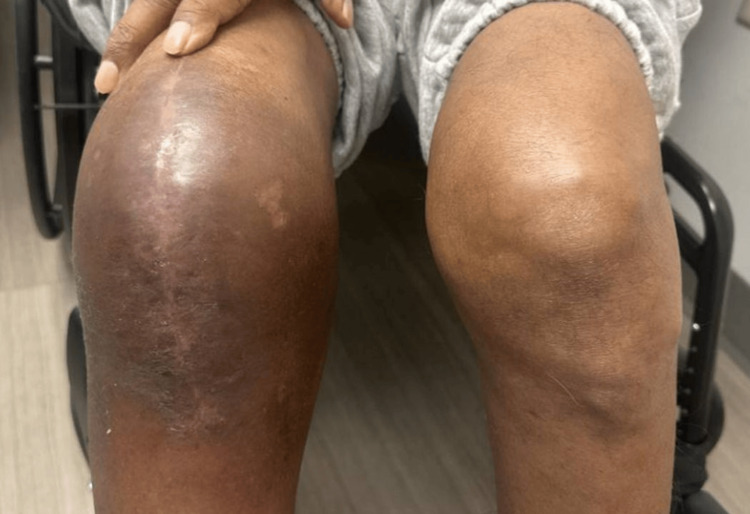
Bilateral knees one month after the patient’s discharge from his initial hospitalization while visiting the outpatient clinic.

One month following discharge, he was seen in the outpatient clinic and was recovering (Figure 4).

**Figure 5:**
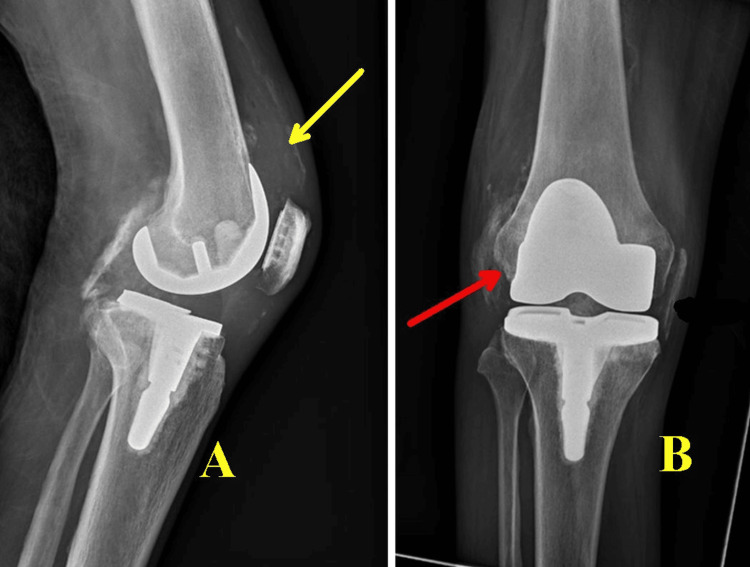
X-rays of the right knee at the time of admission for subsequent hospitalization. They demonstrate intact hardware along with soft tissue swelling in the suprapatellar region in A (yellow arrow) and new mild cortical irregularity in the medial femoral condyle (red arrow), which is suspicious for ongoing infection in B.

**Figure 6:**
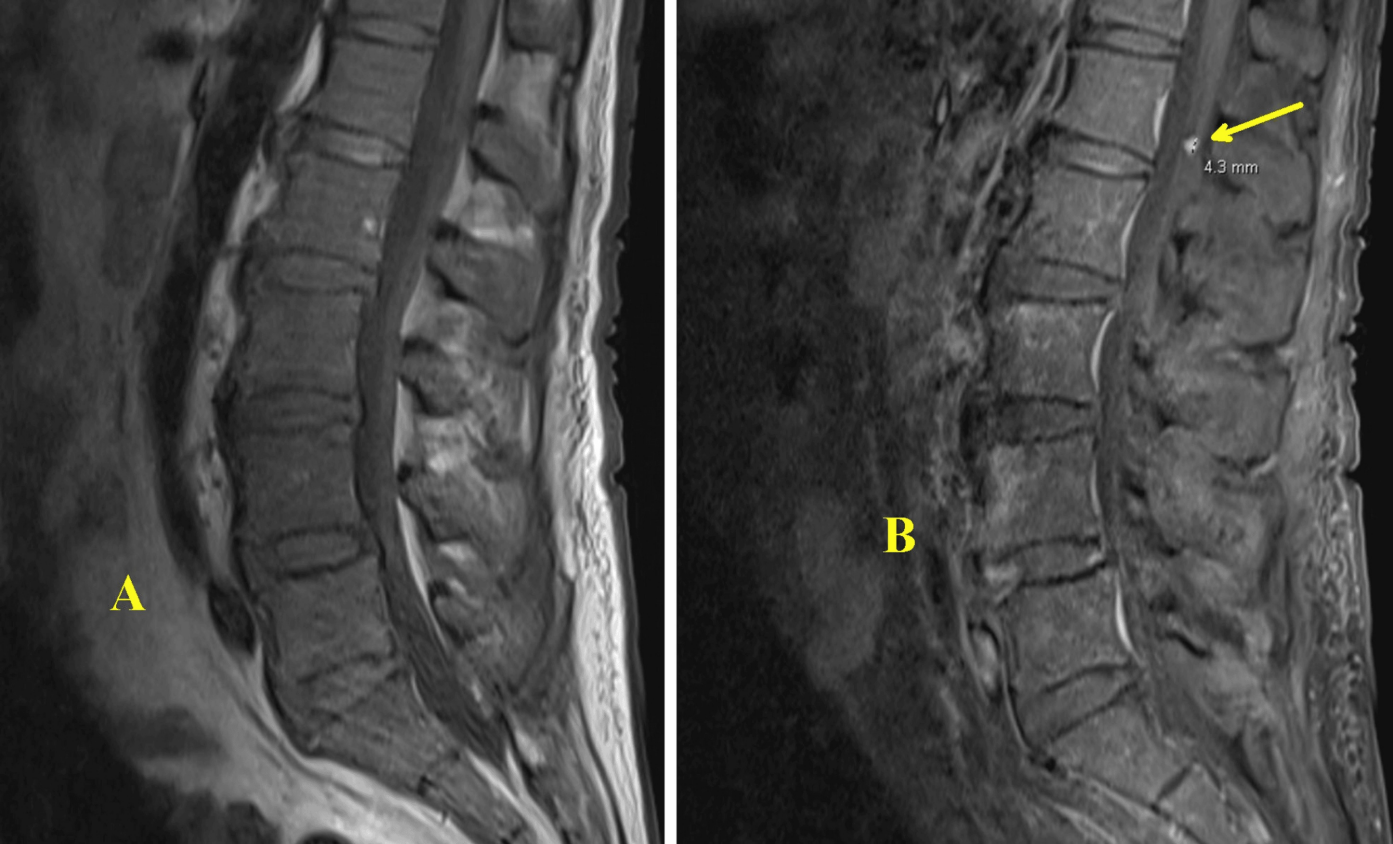
MRI lumbar spine with (A) and without contrast (B) showing a 4.3 mm enhancing nodule in the conus medullaris. Although it is nonspecific, it could represent tuberculoma in the patient’s clinical setting.

**Figure 7:**
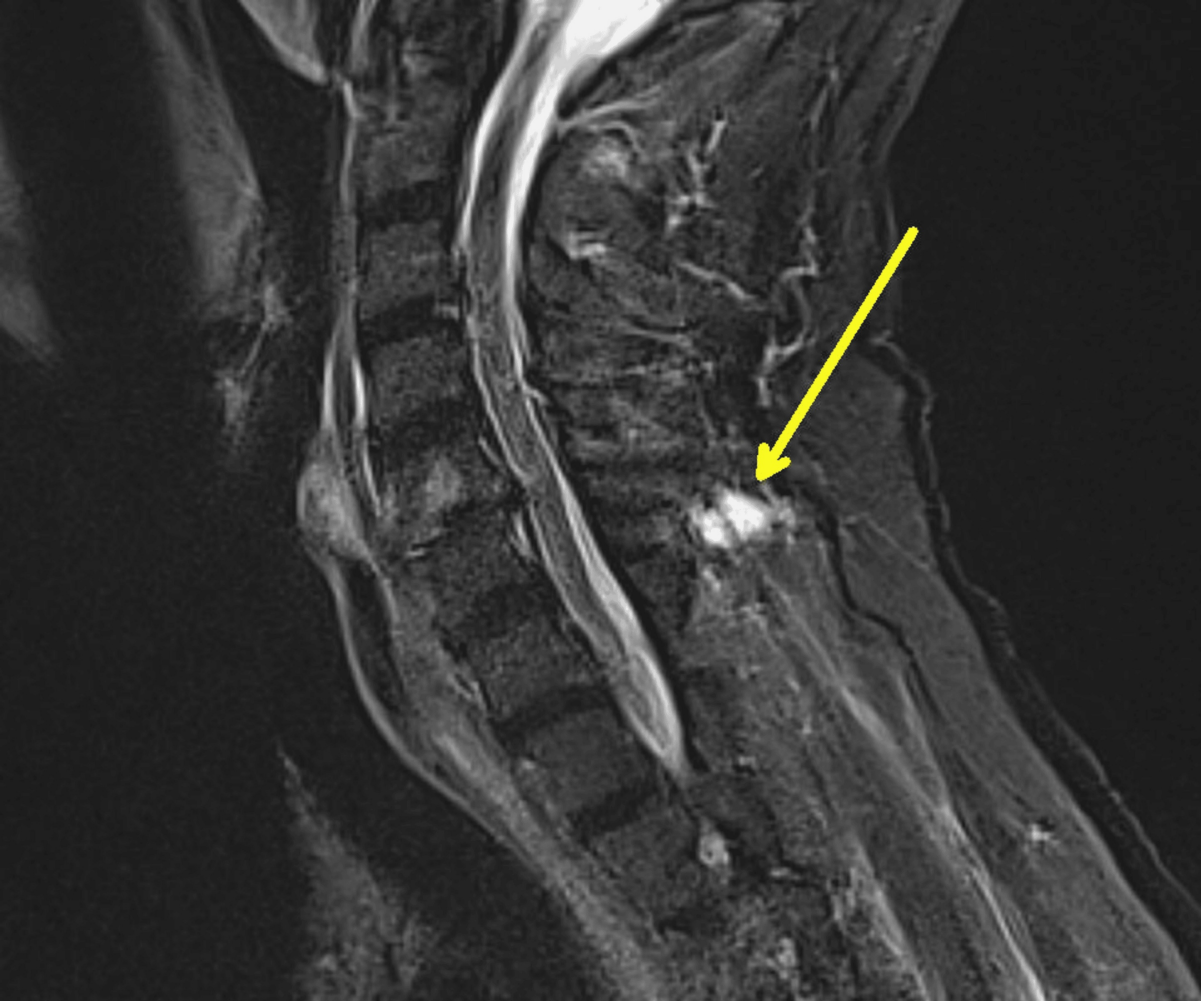
MRI cervical spine with contrast showing nonspecific fluid measuring 1.2 cm adjacent to the C6 spinous process (yellow arrow). This could represent an abscess, seroma, or hematoma.

The patient returned to the hospital two months later with symptoms of general weakness, right knee pain, and fever. An X-ray of the right knee (Figure 5) raised concerns for infection. An arthrocentesis was performed which demonstrated 10 mL of bloody aspirate with < 2,000 red blood cells/uL, 308 white blood cells/uL, 36% neutrophils, 39% lymphocytes, and 23% monocytes/macrophages, inconsistent with reinfection. Synovial cultures and AFB stain/culture were also negative. Magnetic resonance imaging (MRI) of the entire spine, with and without contrast, showed extensive degenerative changes, a 4.3 mm inflammation on the conus medullaris (Figure 6), and inflammation of the left C7 facet joint (Figure 7).

Spinal lesions could not be biopsied by interventional radiology because of their location. Concern for disseminated, suspected multidrug-resistant tuberculosis arose due to continued clinical decline despite appropriate RIPE therapy, combined with spinal MRI abnormalities and the absence of an alternative infectious diagnosis. Considering this, levofloxacin and linezolid were added to his antimicrobial regimen. He underwent a lumbar puncture and a repeat arthrocentesis which were both inconclusive. The arthrocentesis demonstrated 1 mL of bloody aspirate, which was insufficient for cell counts, but cultures were negative. The lumbar puncture demonstrated 2 white blood cells/uL, 0 red blood cells/uL, a glucose of 93 mg/dL, and a protein of 25 mg/dL. The cerebrospinal fluid cultures, AFB stain/culture, and meningitis polymerase chain reaction panel were all negative. The patient was cleared for discharge after a 14-day hospital stay. Karius testing at this time was again negative. 

**Figure 8:**
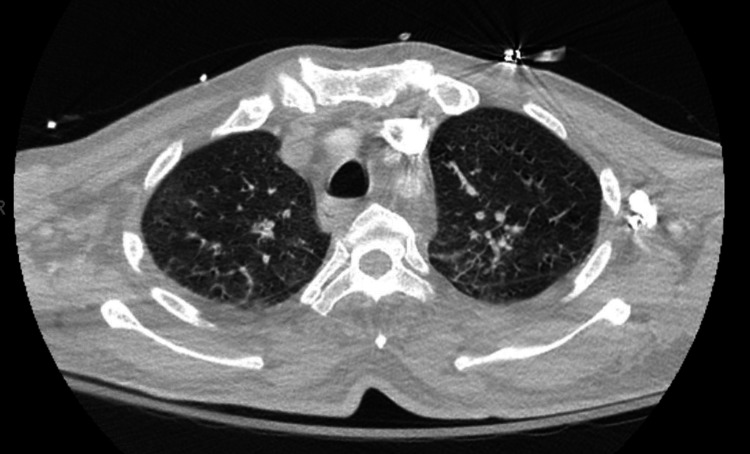
CT chest showing new bilateral upper lobe ground glass interstitial opacities.

The patient returned to the hospital one week later with fevers, rigors, and encephalopathy. A CT scan of the chest showed new lung infiltrates (Figure 8).

**Figure 9:**
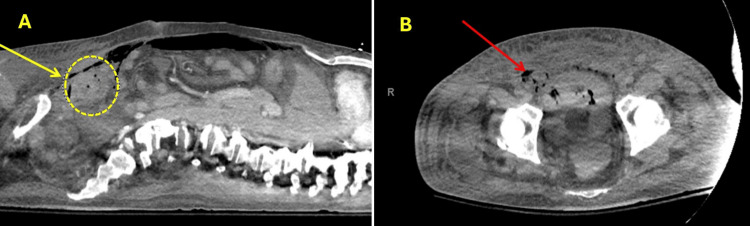
CT abdomen (A: Cross table lateral, B: Transverse), showing extraperitoneal gas and fluid with possible free air in the abdomen (red arrow), anterior abdominal wall soft tissue emphysema, and a questionable small hematoma superior to the bladder (yellow arrow).

He was started on steroids due to concern about immune reconstitution inflammatory syndrome in the setting of presumed disseminated TB. The hospitalization was complicated by gross hematuria after a Foley catheter was placed, which led to cystoscopy with irrigation to remove blood clots from the bladder-an attempt that was unsuccessful. The patient then developed respiratory failure after returning from the operating room and needed to remain intubated and be transferred to the intensive care unit. He also experienced further blood loss and hypotension requiring vasopressors. A CT scan of the abdomen (Figure 9) at that time revealed extraperitoneal gas and fluid, with possible free air in the abdomen, raising concern for bladder injury with irrigation or urine extravasation and a large hematoma or mass above the bladder. The patient underwent a combined procedure with general surgery and urology, which confirmed bladder rupture with extensive blood clots and extravasation into the peritoneal cavity; the bladder was repaired. He was started on broad-spectrum antibiotics and continuous bladder irrigation. Linezolid was discontinued due to worsening thrombocytopenia. Subsequently, the patient developed renal failure. The family decided to remove him from the ventilator and begin comfort measures, and the patient passed away shortly thereafter. 

## Discussion

TB-PJIs are frequently misdiagnosed or discovered late due to their rarity and indolent clinical course. In this case, persistent fevers and negative cultures delayed TB consideration until next-generation sequencing was pursued. Although next-generation sequencing provided a diagnosis, sequencing-based diagnostics (including targeted 16S and metagenomic approaches) have important limitations: results can be influenced by contamination or cross-sample signal, false positives are possible, send-outs and cost/availability may limit access in routine practice [8]. Accordingly, sequencing results should be interpreted in a clinical context and, when feasible, corroborated with pathology or mycobacterial culture. In culture-negative PJIs, it may provide critical diagnostic information when conventional methods fail. Literature suggests that AFB stains and even synovial cultures are often negative in TB joint infections, necessitating molecular diagnostics [4]. Osteoarticular TB may have low organism burden, and synovial fluid testing can be falsely negative; tissue culture may be required yet can still be insensitive, contributing to delayed diagnosis and limited ability to perform drug sensitivity testing [9]. This is a limitation highlighted in this case.

Most patients with TB-PJIs lack prior TB history. In a review of 155 cases, only 42 (27%) had documented TB infection prior to the index case [7]. This patient’s lack of pulmonary symptoms or immunosuppression contributed to diagnostic delay, despite TB exposure risk from his country of origin [10]. First-line therapy for TB-PJIs includes RIPE for 9-12 months, often combined with surgical debridement or prosthesis exchange [5]. In suspected multidrug-resistant TB (MDR-TB), defined as resistance to at least isoniazid and rifampin, second-line therapies such as levofloxacin and linezolid are needed, significantly complicating care due to increased toxicity and lengthened treatment. Concern for disseminated suspected multidrug-resistant tuberculosis arose due to continued clinical decline despite appropriate RIPE therapy, combined with spinal MRI abnormalities and the absence of an alternative infectious diagnosis. Drug levels were not obtained, providing a limitation in determining MDR-TB. While medication adherence was not formally assessed, the patient was discharged to a skilled nursing facility with Department of Health involvement, making nonadherence unlikely. In this patient, treatment interruption due to severe complications and likely suspected MDR-TB contributed to rapid clinical decline.

## Conclusions

TB-PJI is a rare but clinically significant form of PJI that requires high clinical suspicion, especially in the setting of culture-negative PJIs refractory to empiric treatment. Diagnosis is often delayed due to nonspecific presentation and low sensitivity of routine cultures and stains. Treatment is further complicated when multidrug resistance is suspected, as illustrated in this case. Early use of advanced diagnostics and tailored antimicrobial therapy is crucial for improved outcomes.

This case demonstrates several takeaways when clinicians are faced with diagnosing TB-PJIs. The diagnosis can be difficult to make as TB is often a difficult organism to isolate, and patients may not present with pulmonary disease or symptoms typical of TB infection. While TB exposure and risk factors may be important clues to the diagnosis, one or both may not be present in the patient’s history. Factors including TB-PJI rarity, difficulty isolating the organism, and absence of pulmonary symptoms or known TB exposure make it a seldom considered diagnosis early in PJIs. Treatment often requires prolonged therapy, which can be complicated by treatment toxicities and medical complications. Early recognition and use of next-generation sequencing may be needed to avoid diagnostic delays and minimize complications, particularly when initial diagnostic modalities and treatments fail.

## References

[1] (2025). Global tuberculosis report 2024. https://www.who.int/teams/global-programme-on-tuberculosis-and-lung-health/tb-reports/global-tuberculosis-report-2024.

[2] Golden MP, Vikram HR (2005). Extrapulmonary tuberculosis: an overview. Am Fam Physician.

[3] Malaviya AN, Kotwal PP (2003). Arthritis associated with tuberculosis. Best Pract Res Clin Rheumatol.

[4] Tuli SM (2002). General principles of osteoarticular tuberculosis. Clin Orthop Relat Res.

[5] Berbari EF, Hanssen AD, Duffy MC, Steckelberg JM, Osmon DR (1998). Prosthetic joint infection due to Mycobacterium tuberculosis: a case series and review of the literature. Am J Orthop (Belle Mead NJ).

[6] Benito N, Franco M, Ribera A (2016). Time trends in the aetiology of prosthetic joint infections: a multicentre cohort study. Clin Microbiol Infect.

[7] Auñon A, Salar-Vidal L, Mahillo-Fernandez I (2024). Prosthetic joint infections caused by Mycobacterium tuberculosis complex-an ESGIAI-ESGMYC multicenter, retrospective study and literature review. Microorganisms.

[8] Gu W, Miller S, Chiu CY (2019). Clinical metagenomic next-generation sequencing for pathogen detection. Annu Rev Pathol.

[9] Lo CK, Chen L, Varma S, Wood GC, Grant J, Wilson EW (2021). Management of Mycobacterium tuberculosis prosthetic joint infection: 2 cases and literature review. Open Forum Infect Dis.

[10] Margaretten ME, Kohlwes J, Moore D, Bent S (2007). Does this adult patient have septic arthritis?. JAMA.

